# Flavored Tobacco Use Among Canadian Students in Grades 9 Through 12: Prevalence and Patterns From the 2010–2011 Youth Smoking Survey

**DOI:** 10.5888/pcd11.140094

**Published:** 2014-06-19

**Authors:** Leia M. Minaker, Rashid Ahmed, David Hammond, Steve Manske

**Affiliations:** Author Affiliations: Rashid Ahmed, University of Waterloo, Waterloo, Ontario, and Cancer Care Manitoba, Winnipeg, Manitoba; David Hammond, Steve Manske, University of Waterloo, Waterloo, Ontario.

## Abstract

**Introduction:**

This study examined patterns of use of flavored tobacco products in a nationally generalizable sample of Canadian students in grades 9 through 12 after the implementation of a national ban on certain flavored tobacco products.

**Methods:**

Data from the 2010–2011 Youth Smoking Survey, a nationally generalizable sample of Canadian students in grades 9 through 12 (n = 31,396), were used to examine tobacco product use. Logistic regression models were used to examine differences in use of flavored tobacco products (cigarettes, pipes, little cigars or cigarillos, cigars, roll-your-own cigarettes, bidis, smokeless tobacco, water pipes, and blunt wraps) by sociodemographic and regional characteristics.

**Results:**

Approximately 52% of young tobacco users used flavored products in the previous 30 days. Flavored tobacco use varied by product type and ranged from 32% of cigarette smokers reporting menthol smoking to 70% of smokeless tobacco users reporting using flavored product in the previous 30 days. The percentage of last-30-day users who used flavored tobacco was significantly higher in Quebec than in Ontario and significantly higher among youths who received weekly spending money than among those who received no money.

**Conclusion:**

More than half of tobacco users in grades 9 through 12 in Canada use flavored tobacco, despite a national ban on certain flavored tobacco products.

## Introduction

Tobacco use is the leading cause of preventable death in Canada and globally (1), despite dramatic declines in tobacco use rates among Canadian adults and youths during the past 3 decades (2). Flavored tobacco is among the product innovations used by tobacco companies to promote the appeal of their products to young people (3). Manufacturers have used menthol for at least 5 decades to reduce the harshness of cigarette smoke (4). More recent industry innovations in flavor technology resulted in the marketing of candy-, fruit-, and alcohol-flavored cigarettes (5). When R.J. Reynolds Tobacco Company introduced flavored Camel brand extensions, it experienced a sales volume increase of 9.8% in the second quarter of 2004 (6). Other tobacco products, including smokeless tobacco, have similarly been flavored to increase sales. In a longitudinal market trend study, flavor was determined to be one of the most influential characteristics driving the growth in smokeless tobacco sales, accounting for 59.4% of the total growth in moist snuff sales from 2005 to 2011 (7). The use of flavorants in tobacco products is widely acknowledged to make the products more palatable and attractive to new users (4,7,8), particularly young people who are considered “high-sensation seekers.” (9) A study examining industry documents found that flavored tobacco had additional “consumer benefits,” including increased social acceptance because of pleasing aromas and aftertaste, increased excitement (including the sharing of flavors), increased smoking enjoyment, and a “high curiosity to try factor” (4). The World Health Organization’s Framework Convention on Tobacco Control states that sweet-flavored cigarettes fall into the category of products likely to create an erroneous impression that the product is less harmful than other tobacco products (9).

Use of flavored tobacco has been inversely associated with age (5,10,11) in part because of selective marketing to youths (3). A review by the US Food and Drug Administration concluded that the weight of the available evidence supports the conclusion that menthol in cigarettes is likely associated with increased rates of initiation and progression to regular cigarette smoking as well as reduced rates of success in smoking cessation (12). A large, longitudinal study showed that youths who start smoking menthol cigarettes are at greater risk of progression to regular smoking and to nicotine dependence than are youths who start smoking regular cigarettes (13). One prospective study found that youths aged 11 to 15 who recognized Newport (menthol) cigarettes in an advertisement at baseline were more likely to initiate smoking at follow-up 1 year later (after the study adjusted for pertinent covariates) (14). Qualitative research with young adults suggests that the flavor elements in certain tobacco products are particularly appealing to adolescents (15).

An increasing number of jurisdictions have restricted the use of flavors in tobacco products. In 2010, Canada’s Bill C-32 went into effect, prohibiting the sale of cigarettes, little cigars and cigarillos, and blunt wraps that weigh less than 1.4 g and that contain certain additives, including most flavoring agents (excluding menthol); the bill also prohibits the sale of these products in units fewer than 20 items (16). Bill C-32 did not cover all tobacco products: smokeless tobacco, hookah, and other products were untouched. In many cases, manufacturers increased the weight of their products to more than 1.4 g to avoid the law’s requirements. These “new” products, which look identical to the old products, continue to contain added flavors.

The use of flavored tobacco has not been examined in a nationally generalizable sample of Canadian youths. The objective of this study was to examine the use of flavored tobacco products, including menthol cigarettes, among a nationally generalizable sample of Canadian students in grades 9 through 12 after the enactment of Bill C-32. The study also aimed to identify sociodemographic and geographic correlates of flavored tobacco use.

## Methods

### Study design

The 2010–2011 Youth Smoking Survey (YSS) is a nationally generalizable school-based, paper-and-pencil survey that is used to measure the determinants of smoking behavior among youths (17). The target population was students in grades 6 through 12 (ages 11–17) at public and private schools (n = 426) in 9 provinces. Those residing in New Brunswick, Yukon, Nunavut, and Northwest Territories and those living in institutions or on First Nations reserves were excluded. Estimates of smoking rates (based on 2008–2009 YSS) did not vary when data from 1 small East Coast province (New Brunswick) were excluded in 2010–2011. Surveys were pilot tested to assess the logic and student understanding of the questions. Approximately 76% of respondents participated with passive parental permission, and 24% participated with active parental permission. The YSS was administered during class time, and participants were not remunerated. Survey development, design, weights, response rates, and data collection protocol for the 2008 YSS have been published (18). On average, 56% of schools that were approached participated (range, 39% in British Columbia to 100% in Newfoundland), and 73% of eligible students completed questionnaires. Preliminary analyses indicated that tobacco use among elementary school students was not prevalent enough to provide stable estimates; therefore, our analyses were restricted to the secondary school portion of 2010–2011 YSS, which was administered to 31,396 youths in grades 9 through 12 (approximately ages 14–17) attending schools from 9 (of 10) Canadian provinces. (In Quebec, secondary school ends at grade 11.) We combined data from Newfoundland, Nova Scotia and Prince Edward Island to create the category “Atlantic.” Data were collected from October 2010 through June 2011, after implementation of Bill C-32 in July 2010. Data were analyzed in 2013. This study was approved by the University of Waterloo Human Research Ethics Committee, the Health Canada Research Ethics Board, and appropriate school board and public health ethics committees.

### Measures and data sources

Relevant, dichotomous outcomes included “ever use” and “use in the last 30 days” of cigarettes, “any” tobacco product, and flavored tobacco products. Students who responded yes to the question “Have you ever tried cigarette smoking, even just a few puffs?” were defined as ever users of cigarettes. Ever users of other forms of tobacco were those who reported ever trying at least 1 of the following: cigarettes; pipe tobacco; little cigars (plain or flavored) or cigarillos; cigars (plain or flavored); roll-your-own cigarettes (tobacco only); bidis (little cigarettes that are hand-rolled in leaves, tied with a string at the ends, and come in different flavors); smokeless tobacco (chewing tobacco, pinch, snuff, or snus); a water pipe to smoke tobacco (also known as hookah, sheesha, narg-eelay, hubble-bubble, or gouza); or blunt wraps (a sheet or tube made of tobacco used to roll cigarette tobacco). Ever users of flavored tobacco were defined as participants who responded yes to the question “Have you ever used flavored tobacco products (menthol, cherry, strawberry, vanilla, etc.)?” Last-30-day users of cigarettes reported smoking at least 1 cigarette on at least 1 day of the last 30 days. Last-30-day users of other forms of tobacco responded yes to at least 1 of the response options to the question “In the last 30 days, did you use any of the following?” The response options were pipe tobacco; little cigars or cigarillos; cigars; roll-your-own cigarettes; bidis; smokeless tobacco; a water pipe to smoke tobacco; and blunt wraps. Last-30-day users of flavored tobacco responded yes to at least 1 of the response options to the question “In the last 30 days, did you use any of the following flavored tobacco products?” Response options were menthol cigarettes; flavored little cigars or cigarillos; flavored cigars; flavored bidis; flavored smokeless tobacco; flavored tobacco in a hookah (water pipe). Student reports of last-30-day use of flavored tobacco products were considered valid only if they had also responded yes to the previous question assessing last-30-day use of that particular product. For example, menthol smoking in the last 30 days was considered valid only if the student also reported smoking at least 1 cigarette in the last 30 days. Similarly, flavored bidi smoking was considered valid only if the student also reported smoking bidis in the last 30 days. Therefore, estimates of the proportion of tobacco users using flavored products in the last 30 days are likely conservative.

Independent variables included the respondent’s sex, grade (9–12), province, self-reported race/ethnicity (white, black, Asian, Aboriginal, Latin American, or “other”), and the amount of weekly spending money received. Weekly spending money was categorized as none, $1 to $10, $11 to $40, and more than $40 (in Canadian currency). Two covariates were also included in analyses: whether any family members smoke (yes or no) and the number of closest friends who smoke (none; 1; 2–4; and >4). The 2008–2009 YSS survey did not ask about flavored tobacco product use, so we could not perform analyses on time trends.

### Statistical analysis

Survey weights were used to adjust for sample selection (school and class levels), nonresponse (school, class, and student levels), and poststratification of the sample population relative to grade and sex distribution in the total population. Descriptive statistics were used to show the use of plain and flavored tobacco by sex, grade, province of residence, self-reported race/ethnicity, and weekly spending money. Proportions were calculated as percentages to show use of tobacco and flavored tobacco in the last 30 days by product type. Finally, 3 logistic regression models were fitted to examine independent variables related to the odds of using various forms of tobacco in the last 30 days. Independent variables included sex, grade, province of residence, self-reported race/ethnicity, and weekly spending money. Outcomes in the 3 models were chosen to represent 1) overall flavored tobacco use; 2) flavored tobacco use represented by menthol cigarette smoking (because menthol was excluded from Bill C-32); and 3) flavored tobacco use represented by little cigars and cigarillos (because preliminary results show use of little cigars and cigarillos to be second only to cigarettes in prevalence). Model 1 examined last-30-day use of any type of flavored tobacco product among all current tobacco users. Model 2 examined the subset of flavored use represented by menthol cigarettes among all cigarette users, and model 3 examined the subset of flavored use represented by little cigars or cigarillos among all little cigar or cigarillo users. Variables were entered into the final model if they were significantly associated with the outcome at a *P* value of less than .05 for any level of the variable. Full models initially consisted of main exposure, outcome, and covariates. Assumptions of logistic regressions (eg, sufficient sample size for single cell counts) were checked, and goodness-of-fit tests were used to check model fit. A backward elimination strategy was used to evaluate each covariate in the presence of others. Covariates were removed individually when the change-in-estimate of effect measure was less than 10%. The final models showed the measure of association between an independent variable of interest and outcome. Logistic regressions were conducted by using PROC SURVEYLOGISTIC in SAS 9.3.2 (SAS Institute Inc, Cary, North Carolina).

## Results

In 2010–2011, 41.7% of respondents reported ever using any form of tobacco, and 36.6% reported ever smoking cigarettes (Table 1). Of ever users of any form of tobacco, 59.0% had ever used flavored tobacco. Among respondents reporting any form of tobacco use in the last 30 days, 51.8% used flavored tobacco in the same period. The percentage of ever users who had used flavored tobacco ranged from 52.7% in grade 9 to 64.3% in grade 12. Ontario had the lowest prevalence of last-30-day use of flavored tobacco as a percentage of overall tobacco use (46.3%) and Quebec had the highest (59.3%). Last-30-day use of flavored tobacco as a percentage of overall tobacco use was highest among self-reported black respondents (57.9%) and lowest among self-reported “other” race/ethnicities (34.7%) and Asian youths (35.6%).

**Table 1:** Weighted Prevalence of Ever Having Used or Used in the Last 30 Days Plain or Flavored Tobacco, Grades 9–12, Canadian Youth Smoking Survey, 2010–2011

Characteristic	N (%)^a^	Ever Used, Weighted Prevalence, %				Used in Last 30 Days, Weighted Prevalence, %				
		Cigarette Smoking^a^	Any Form of Tobacco^b^	Any Form of Flavored Tobacco^c^	Flavored Tobacco Among Tobacco Users^d^	Cigarette Smoking^e^	Any Form of Tobacco^f^	Any Form of Flavored Tobacco^g^	Flavored Tobacco Among Tobacco Users^h^	Menthol Smoking Among Cigarette Smokers^i^
**Canada**	31,396 (100)	36.6	41.7	25.2	59.0	14.4	19.9	10.3	51.8	31.7
**Sex**										
Female	15,707 (50)	34.4	38.4	22.6	58.3	12.9	16.1	8.3	51.5	32.0
Male	15,689 (50)	38.7	44.9	27.5	59.6	15.9	23.5	12.2	52.0	31.5
**Grade**										
9	8,196 (26)	26.7	29.6	15.9	52.7	9.8	12.8	6.6	51.3	31.6
10	8,706 (28)	33.7	37.8	22.0	55.8	12.3	17.4	8.9	51.2	32.9
11	8,059 (26)	39.0	46.3	28.2	59.9	15.5	21.6	12.1	56.1	33.9
12	6,435 (20)	47.7	53.8	35.1	64.3	20.5	28.3	13.8	48.8	29.2
**Province**										
Ontario	6,412 (20)	29.7	34.6	19.0	53.4	11.0	16.3	7.5	46.3	28.2
Atlantic^j^	8,906 (28)	45.0	48.7	28.8	58.8	20.0	24.7	12.0	48.7	36.6
Quebec	1,502 (5)	42.7	50.0	34.5	66.8	14.8	20.6	12.3	59.3	26.0
Manitoba	5,225 (17)	36.2	39.7	20.1	49.9	13.2	17.7	8.8	49.4	33.6
Saskatchewan	1,608 (5)	53.0	57.1	35.7	61.2	27.8	33.7	18.2	54.0	35.7
Alberta	2,488 (8)	40.1	44.4	27.2	60.6	17.8	23.4	13.1	55.9	36.6
British Columbia	5,255 (17)	40.9	46.0	28.9	62.4	16.9	23.0	12.2	53.0	36.0
**Race/ethnicity**										
White	23,554 (76)	38.6	44.0	27.4	61.0	15.2	21.0	11.4	54.3	31.3
Black	1,019 (3)	32.2	40.2	25.1	60.7	15.9	25.0	14.5	57.9	49.0
Asian	3,409 (11)	20.6	23.3	10.6	44.2	7.3	9.5	3.4	35.6	22.2
Aboriginal	1,272 (4)	72.0	74.2	44.1	59.1	36.3	41.6	19.2	46.1	32.1
Latin American	528 (2)	46.9	50.8	29.6	56.4	14.8	26.9	14.0	52.0	60.0
Other	1,379 (4)	28.1	33.8	18.9	52.4	7.6	12.7	4.4	34.7	24.7
**Weekly spending money**										
No money	5,025 (19)	27.3	31.9	17.9	54.2	9.2	12.6	5.6	44.2	27.9
$1–$10	4,070 (16)	26.7	31.0	17.8	56.3	10.7	14.3	8.0	56.1	35.8
$11–$40	9,276 (35)	38.8	43.0	25.4	58.2	14.7	20.9	10.9	52.3	32.0
>$40	7,813 (30)	49.1	56.2	36.2	63.0	21.7	29.4	16.0	54.6	32.3

a Percentage of respondents who reported yes to the question “Have you ever tried cigarette smoking, even just a few puffs?”

b Percentage of respondents who identified at least 1 option for the question “Have you ever tried any of the following?” Response options included the following: pipe tobacco, cigarillos or little cigars (plain or flavored); cigars (plain or flavored); roll-your-own cigarettes (tobacco only); bidis (little cigarettes that are hand-rolled in leaves, tied with a string at the ends, and come in different flavors); smokeless tobacco (chewing tobacco, pinch, snuff, or snus); a water pipe to smoke tobacco (also known as hookah, sheesha, narg-eelay, hubble-bubble, or gouza); or blunt wraps (a sheet or tube made of tobacco used to roll cigarette tobacco).

c Percentage of respondents who reported yes to the question “Have you ever used flavored tobacco products (menthol, cherry, strawberry, vanilla, etc.)?”

d Percentage of respondents who reported using a flavored tobacco product among those who reported ever using any tobacco product.

e Percentage of respondents who reported smoking at least 1 cigarette on at least 1 day of the last 30 days.

f Percentage of respondents who identified at least 1 option for the question “In the last 30 days, did you use any of the following?” Response options included the following: pipe tobacco; little cigars or cigarillos; cigars; roll-your-own cigarettes; bidis; smokeless tobacco; a water pipe to smoke tobacco; and blunt wraps.

g Percentage of respondents who reported using at least 1 of the following products in the last 30 days: menthol cigarettes; flavored little cigars or cigarillos; flavored cigars; flavored bidis; flavored smokeless tobacco; flavored tobacco in a hookah (water pipe).

h Percentage of respondents who reported using a flavored tobacco product in the last 30 days among those who reported using any tobacco product in the last 30 days.

i Percentage of respondents who reported smoking menthol cigarettes in the last 30 days among those who reported smoking cigarettes in the last 30 days.

j Includes data from Newfoundland, Nova Scotia, and Prince Edward Island.

Almost one-third (31.7%) of respondents who smoked cigarettes in the previous 30 days smoked menthol cigarettes (Table 2). Not counting cigarettes, use of little cigars or cigarillos in the last 30 days accounted for the highest prevalence of both plain or flavored products (7.8%) and flavored products only (5.1%). Excluding menthol cigarettes, at least half of all respondents who reported using various tobacco products in the last 30 days reported using flavored tobacco products (ranging from 49.8% of cigar users to 69.9% of smokeless tobacco users).

**Table 2:** Last-30-Day Use of Tobacco and Flavored Tobacco by Product Type, Grades 9 Through 12, Canadian Youth Smoking Survey, 2010–2011

Last-30-day use	Used Tobacco in Last 30 Days, % (n)^a^		Last-30-Day Users Who Used Flavored Products in Last 30 Days, %
	Any Tobacco Product (Plain or Flavored)^b^	Flavored Tobacco Product Only^c^	
Cigarettes (menthol)^d^	14.5 (5,035)	4.6 (1,725)	31.7
Pipe tobacco	2.6 (856)	NA	NA
Little cigars and cigarillos	7.8 (2,389)	5.1 (1,979)	65.4
Cigars (not little cigars and cigarillos)	5.0 (1,697)	2.5 (853)	49.8
Roll-your-own cigarettes	3.7 (1,473)	NA	NA
Bidis	0.9 (345)	0.6 (185)	66.6
Smokeless tobacco	2.5 (895)	1.7 (569)	69.9
Water pipe	3.8 (1,002)	2.1 (530)	54.4
Blunt wraps	2.8 (1,016)	NA	NA

Abbreviations: NA, not applicable; DWR, delayed word recall; BMI, body mass index.

a For all values, weighted percentages are given, and *n* is rescaled to the original sample size.

b Percentage of respondents who identified at least 1 option for the question “In the last 30 days, did you use any of the following?” Response options included the following: pipe tobacco; little cigars or cigarillos; cigars; roll-your-own cigarettes; bidis; smokeless tobacco; a water pipe to smoke tobacco; and blunt wraps.

c Percentage of respondents who reported using at least 1 of the following products in the last 30 days: menthol cigarettes; flavored little cigars or cigarillos; flavored cigars; flavored bidis; flavored smokeless tobacco; flavored tobacco in a hookah (water pipe).

d Menthol cigarette smoking comprises the flavored tobacco-product component of last-30-day use of cigarettes. Students who reported smoking cigarettes within the last 30 days represent the denominator, and students reporting smoking menthol cigarettes in the last 30 days represent the numerator.

In model 1 (Table 3), respondents from Quebec were significantly more likely than respondents from Ontario to use flavored tobacco (OR, 1.8; 95% confidence interval [CI], 1.2–2.6). Respondents who self-reported “other” race/ethnicity were significantly less likely than white respondents to use flavored tobacco (OR, 0.4; 95% CI, 0.2–0.8). Respondents who reported receiving $1 to $10 per week or more than $40 per week in spending money were significantly more likely than respondents who received no spending money to use flavored tobacco in the last 30 days (OR, 1.7; 95% CI, 1.1–2.7 and OR, 1.6; 95% CI, 1.1–2.3, respectively).

**Table 3:** Logistic Regression Analysis of Variables Related to Likelihood (OR [95% CI]) of Use of Various Forms of Tobacco in the Last 30 Days, Grades 9 Through 12, Canadian Youth Smoking Survey, 2010–2011

Predictor	Model 1: Any Form of Flavored Tobacco Use Among All Tobacco Users (N = 6,495)^a^	Model 2: Menthol Cigarette Use Among Cigarette Users (N = 5,035)^b^	Model 3: Flavored Cigarillo or Little Cigar Use Among Cigarillo or Little Cigar Users (N = 2,389)^c^
**Sex**			
Female	1 [Reference]	1 [Reference]	1 [Reference]
Male	1.2 (1.0– 1.6)	1.1 (0.8– 1.4)	0.9 (0.6–1.5)
**Grade**			
9	1 [Reference]	1 [Reference]	1 [Reference]
10	0.8 (0.6–1.4)	0.9 (0.5–1.4)	0.8 (0.4–1.6)
11	1.3 (0.9–1.8)	1.0 (0.6–1.5)	1.1 (0.6–2.0)
12	0.9 (0.6–1.4)	0.9 (0.5–1.4)	1.7 (0.9–3.1)
**Province**			
Ontario	1 [Reference]	1 [Reference]	1 [Reference]
Atlantic	1.0 (0.8–1.3)	1.3 (1.0–1.7)	0.8 (0.6–1.2)
Quebec	1.8 (1.2–2.6)	0.9 (0.5–1.6)	0.7 (0.4–1.3)
Manitoba	1.1 (0.8–1.4)	1.3 (0.9–1.9)	0.9 (0.6–1.4)
Saskatchewan	1.4 (1.0–1.9)	1.4 (0.9–2.1)	0.9 (0.6–1.6)
Alberta	1.4 (0.9–2.2)	1.4 (0.8–2.2)	1.1 (0.5–2.3)
British Columbia	1.3 (0.9–1.8)	1.3 (0.8–2.0)	1.2 (0.6–2.1)
**Race/ethnicity**			
White	1 [Reference]	1 [Reference]	1 [Reference]
Black	1.3 (0.8–2.1)	2.1 (1.1–4.0)	1.1 (0.4–2.7)
Asian	0.5 (0.2–1.2)	0.6 (0.2–1.6)	0.7 (0.2–2.5)
Aboriginal	0.7 (0.5–1.0)	0.8 (0.5–1.3)	0.6 (0.3–1.1)
Latin American	0.9 (0.2–3.0)	3.2 (1.1–9.5)	0.5 (0.1–3.2)
Other	0.4 (0.2–0.8)	0.5 (0.2–1.1)	0.8 (0.3–2.5)
**Weekly spending money**			
No money	1 [Reference]	1 [Reference]	1 [Reference]
$1–$10	1.7 (1.1–2.7)	1.4 (0.8–2.8)	0.8 (0.3–2.2)
$11–$40	1.4 (1.0–2.1)	1.2 (0.8–2.0)	1.0 (0.5–1.9)
>$40	1.6 (1.1–2.3)	1.1 (0.7–1.8)	1.4 (0.7–2.5)

Abbreviations: OR, odds ratio; CI, confidence interval.

a Final model 1 included the following variables: sex, province, race/ethnicity, weekly spending money, and number of closest friends smoking.

b Final model 2 included the following variables: sex, grade, province, race/ethnicity, weekly spending money, family member smoking, and number of closest friends smoking. For each exposure, the final model included only variables that appeared to be confounders. For example, the final model 2 for modeling with grade was adjusted for province, race/ethnicity, family member smoking, and number of closest friends smoking.

c For all independent variables but race/ethnicity, final model 3 was adjusted for race/ethnicity only. For race/ethnicity, final model 3 was adjusted for province only.

Model 2 showed that self-identified black and Latin American respondents were significantly more likely than white respondents to smoke menthol cigarettes (OR, 2.1; 95% CI, 1.1–4.0 and OR, 3.2; 95% CI, 1.1–9.5, respectively). Model 3 showed no significant associations between the independent variables and flavored cigarillo use.

## Discussion

The rate of using flavored tobacco products is high among Canadian students in grades 9 through 12 after the enactment of Bill C-32. Our study contributes 3 major findings to knowledge about tobacco use in Canada. First, almost one-third (32%) of smokers in our study reported smoking menthol in the last 30 days. Second, more than half (52%) reported using flavored products in the last 30 days. Third, although rates of overall flavored tobacco use and flavored cigarillo use did not vary by race/ethnicity, self-reported black youths were more than 2 times as likely as white youths to smoke menthol cigarettes, and Latin American youths were more than 3 times as likely as white youths to smoke menthol cigarettes.

First, our study showed a high prevalence of menthol cigarette use among young smokers, consistent with data from the United States (11,19). Menthol flavoring was explicitly excluded from Bill C-32 yet has clear implications for uptake of tobacco use among youths (4,8,20). The International Tobacco Control study showed in 2006 that approximately 5% of Canadian adult smokers “usually” smoke menthol cigarettes (21). Health Canada reported that only 2% of Canadian smokers smoke menthol cigarettes, although the source of the Health Canada data is not clear (22).

Although “usual” smokers and “last-30-day” smokers are not directly comparable, the 5% of adult smokers in Canada who usually smoke menthol cigarettes (21) contrasts with the 32% of young smokers in our study who reported smoking menthol cigarettes in the last 30 days. The 5% of participants in our overall sample who smoked menthol cigarettes in the last 30 days is comparable to the 4% of students aged 12 to 17 years who reported smoking menthol cigarettes in the past month in the United States (23). More than 30% of American smokers older than 25 smoke menthol cigarettes (11).

Second, our study showed high overall rates of flavored tobacco use; more than half of the tobacco users in our study reported using flavored products in the last 30 days. Use of flavored little cigars was similar in the United States and Canada in 2010–2011; 4% of US students in grades 6 through 12 (19) and 5% of Canadian students in grades 9 through 12 smoke flavored little cigars. These data are not completely comparable because of the different age ranges. Of students who reported using smokeless tobacco in the last 30 days in our study, 70% reported using flavored smokeless tobacco, higher than the rate of flavored smokeless tobacco use among adult US smokeless tobacco users (59%) (24). Half of students who smoked cigars in the last 30 days smoked flavored cigars; this rate is slightly lower than the national prevalence (57%) of flavored cigar use among adult cigar smokers (aged 18–24) in the United States (25). Flavored tobacco use as a proportion of overall tobacco use varied by province, with a low of 46% in Ontario to a high of 59% in Quebec. 

Although ours was not a pre–post study design, data from the 2008–2009 YSS indicate that 12.9% of Canadian students in grades 9 through12 used cigarillos in the last 30 days (26). Our study data, which were collected after the sale of sweet-flavored cigarillos was prohibited by Bill C-32, indicate that 8% of youths used cigarillos. The drop in little cigar and cigarillo use between survey cycles may indicate an effect of Bill C-32. However, in 2010–2011, 65% of youths who smoked little cigars and cigarillos reported using flavored versions in the previous 30 days, and between 50% and 70% of youths who used alternate forms of tobacco (other than cigarettes) in the previous 30 days reported using flavored products, reflecting the limited ability of Bill C-32 to restrict flavored tobacco use among youth. Expanding the ban on flavors to all tobacco products, including all cigarillos, smokeless tobacco, bidis, and water pipes, may further reduce tobacco use rates among youths. Although cigarette smoking is the most common form of tobacco use among youths and progress has been made to decrease rates of use, efforts to reduce tobacco use require more refinement (27).

Third, our study showed that self-reported black and Latin American respondents were significantly more likely than white respondents to smoke menthol cigarettes. Ours is the first study to provide evidence of racial/ethnic disparities in menthol smoking among Canadian students in grades 9 through 12 and contributes to a small but growing literature on racial/ethnic disparities in smoking among youths in Canada (28–30). Menthol cigarette marketing to African Americans has been well-documented in the media (31) and in neighborhoods (32) in the United States. Despite differences in tobacco marketing regulations between the 2 nations, our finding that black and Latin American students were more likely to smoke menthol cigarettes mirrors recent trends in the United States (11).

Our study provides evidence on use of flavored tobacco among youths in Canada, using a large, nationally generalizable sample of students in grades 9 through 12, but it has several limitations. Because the study was cross-sectional, no causation can be inferred. Because the YSS is a school-based survey, youths who do not attend mainstream schools were excluded from our sample. Similarly, Aboriginals living on reserves and youths living in Canada’s 3 territories were also excluded. Finally, use of novel tobacco products such as strips and orbs were not assessed, and therefore both overall tobacco use and flavored tobacco use may be underestimated.

Our study suggests that menthol cigarettes and other flavored tobacco products have particular appeal among youths. The US Food and Drug Administration is considering new regulations on menthol tobacco products. Legislation that prohibits flavored tobacco has been announced in numerous Canadian provinces, including Alberta (similar to Hawaii’s Bill 2222 [33], legislation to prohibit flavors, including menthol, in tobacco products) (34) and Ontario (35). Future research should examine the effect of provincial legislation on both rates of flavored and overall tobacco use in different provinces.

## References

[1] World Health Organization. WHO report on the global tobacco epidemic 2013. http://www.who.int/tobacco/global_report/2013/en/index.html. Accessed August 13, 2013.

[2] Health Canada. Canadian Tobacco Use Monitoring Survey (CTUMS) 2011. http://www.hc-sc.gc.ca/hc-ps/tobac-tabac/research-recherche/stat/ctums-esutc_2011-eng.php. Accessed August 13, 2013.

[3] Lewis MJ , Wackowski O . Dealing with an innovative industry: a look at flavored cigarettes promoted by mainstream brands. Am J Public Health 2006;96(2):244–51. 10.2105/AJPH.2004.061200 16380563 PMC1470487

[4] Carpenter CM , Wayne GF , Pauly JL , Koh HK , Connolly GN . New cigarette brands with flavors that appeal to youth: tobacco marketing strategies. Health Aff (Millwood) 2005;24(6):1601–10. 10.1377/hlthaff.24.6.1601 16284034

[5] Klein SM , Giovino GA , Barker DC , Tworek C , Cummings KM , O’Connor RJ . Use of flavored cigarettes among older adolescent and adult smokers: United States, 2004–2005. Nicotine Tob Res 2008;10(7):1209–14. 10.1080/14622200802163159 18629731

[6] Berk CC . RJ Reynolds earnings surged in second quarter. Wall Street Journal 2004 Aug 3. http://online.wsj.com/news/articles/SB109145167056680434.

[7] Delnevo CD , Wackowski OA , Giovenco DP , Manderski MTB , Hrywna M , Ling PM . Examining market trends in the United States smokeless tobacco use: 2005–2011. Tob Control 2014. 23(2):107–12. 10.1136/tobaccocontrol-2012-050739 23117999 PMC3604094

[8] Kreslake JM , Wayne GF , Connolly GN . The menthol smoker: tobacco industry research on consumer sensory perception of menthol cigarettes and its role in smoking behavior. Nicotine Tob Res 2008;10(4):705–15. 10.1080/14622200801979134 18418792

[9] Manning KC , Kelly KJ , Comello ML . Flavoured cigarettes, sensation seeking and adolescents’ perceptions of cigarette brands. Tob Control 2009;18(6):459–65. 10.1136/tc.2009.029454 19700436

[10] Hersey JC , Wen Ng S , Nonnemaker JM , Mowery P , Thomas KY , Vilsaint MC , Are menthol cigarettes a starter product for youth? Nicotine Tob Res 2006;8(3):403–13. 10.1080/14622200600670389 16801298

[11] Giovino GA , Villanti AC , Mowery PD , Sevilimedu V , Niaura RS , Vallone DM , Differential trends in cigarette smoking in the USA: is menthol slowing progress? Tob Control 2013. 10.1136/tobaccocontrol-2013-051159 23997070

[12] Food and Drug Administration. Preliminary scientific evaluation of the possible public health effects of menthol versus nonmenthol cigarettes. http://www.fda.gov/downloads/ScienceResearch/SpecialTopics/PeerReviewofScientificInformationandAssessments/UCM361598.pdf. Accessed August 19, 2013.

[13] Nonnemaker J , Hersey J , Homsi G , Allen J , Vallone D . Initiation with menthol cigarettes and youth smoking uptake. Addiction 2013;108(1):171–8. 10.1111/j.1360-0443.2012.04045.x 22862154

[14] Dauphinee AL , Doxey JR , Schleicher NC , Fortmann SP , Henriksen L . Racial differences in cigarette brand recognition and impact on youth smoking. BMC Public Health 2013;13:170. 10.1186/1471-2458-13-170 23442215 PMC3586353

[15] Choi K , Fabian L , Mottey N , Corbett A , Forster J . Young adults’ favorable perceptions of snus, dissolvable tobacco products, and electronic cigarettes: findings from a focus group study. Am J Public Health 2012;102(11):2088–93. 10.2105/AJPH.2011.300525 22813086 PMC3469759

[16] Bill C-32. An act to amend the Tobacco Act, SC 2009. http://www.parl.gc.ca/About/Parliament/LegislativeSummaries/bills_ls.asp?lang=E&ls=c32&Parl=40&Ses=2&source=library_prb. Accessed August 14, 2013.

[17] Propel Centre for Population Health Impact. Youth Smoking Survey 2012–2013. http://www.yss.uwaterloo.ca/index.cfm?section=1001&page=248. Accessed August 15, 2013.

[18] Elton-Marshall T , Leatherdale ST , Manske SR , Wong K , Ahmed R , Burkhalter R . Research methods of the Youth Smoking Survey (YSS). Chronic Dis Inj Can 2011;32(1):47–54. 22153176

[19] King BA , Tynan MA , Dube SR , Arrazola R . Flavored-little-cigar and flavored-cigarette use among U.S. middle and high school students. J Adolesc Health 2014;54(1):40–6. 10.1016/j.jadohealth.2013.07.033 24161587 PMC4572463

[20] Giovino GA , Sidney S , Gfroerer JC , O’Malley PM , Allen JA , Richter PA , Epidemiology of menthol cigarette use. Nicotine Tob Res 2004;6(Suppl 1):S67–81. 10.1080/14622203710001649696 14982710

[21] Mutti S , Hammond D , Borland R , Cummings MK , O’Connor RJ , Fong GT . Beyond light and mild: cigarette brand descriptors and perceptions of risk in the International Tobacco Control (ITC) Four Country Survey. Addiction 2011;106(6):1166–75. 10.1111/j.1360-0443.2011.03402.x 21481054 PMC4419584

[22] Health Canada. An act to amend the Tobacco Act (2009): Frequently asked questions. http://www.hc-sc.gc.ca/hc-ps/tobac-tabac/legislation/federal/amend_faq-modif-eng.php#q7. Accessed August 19, 2013.

[23] National Survey on Drug Use and Health. The NSDUH report: recent trends in menthol cigarette, 2011. http://www.samhsa.gov/data/2k11/WEB_SR_088/WEB_SR_088.pdf. Accessed March 20, 2014.

[24] Oliver AJ , Jensen JA , Vogel RI , Anderson AJ , Hatsukami DK . Flavored and nonflavored smokeless tobacco products: rate, pattern of use, and effects. Nicotine Tob Res 2013;15(1):88–92. 10.1093/ntr/nts093 22529222 PMC3524058

[25] King BA , Dube SR , Tynan MA . Flavored cigar smoking among U.S. adults: findings from the 2009–2010 National Adult Tobacco Survey. Nicotine Tob Res 2013;15(2):608–14. 10.1093/ntr/nts178 22927687

[26] Leatherdale ST , Rios P , Elton-Marshall T , Burkhalter R . Cigar, cigarillo, and little cigar use among Canadian youth: are we underestimating the magnitude of this problem? J Prim Prev 2011;32(3-4):161–70. 10.1007/s10935-011-0248-6 21809109

[27] Warner KE , Mackay J . The global tobacco disease pandemic: nature, causes, and cures. Glob Public Health 2006;1(1):65–86. 10.1080/17441690500430771 19153895

[28] Czoli CD , Leatherdale ST , Rynard V . Bidi and hookah use among Canadian youth: findings from the 2010 Canadian Youth Smoking Survey. Prev Chronic Dis 2013;10:E73. 10.5888/pcd10.120290 23660115 PMC3664211

[29] Elton-Marshall T , Leatherdale ST , Burkhalter R , Brown KS . Changes in tobacco use, susceptibility to future smoking, and quit attempts among Canadian youth over time: a comparison of off-reserve aboriginal and non-aboriginal youth. Int J Environ Res Public Health 2013;10(2):729–41. 10.3390/ijerph10020729 23429753 PMC3635174

[30] Freedman KS , Nelson NM , Feldman LL . Smoking initiation among young adults in the United States and Canada, 1998–2010: a systematic review. Prev Chronic Dis 2012;9(1):E05. 22172172 PMC3277388

[31] Rising J , Alexander L. Marketing of menthol cigarettes and consumer perceptions. Tob Induc Dis 2011;9(Suppl 1):S2. 10.1186/1617-9625-9-S1-S2 21624148 PMC3102901

[32] Moreland-Russell S , Harris J , Snider D , Walsh H , Cyr J , Barnoya J . Disparities and menthol marketing: additional evidence in support of point of sale policies. Int J Environ Res Public Health 2013;10(10):4571–83. 10.3390/ijerph10104571 24071922 PMC3823340

[33] State of Hawaii. A bill for an act relating to flavored tobacco products. http://www.capitol.hawaii.gov/session2014/bills/SB2222_SD2_.htm. Accessed March 20, 2014.

[34] Bill 206: Tobacco Reduction (Flavoured Tobacco Products) Amendment Act, 2013. http://www.assembly.ab.ca/net/index.aspx?p=bills_status&selectbill=206&legl=28&session=1. Accessed January 20, 2014.

[35] Leslie K . Ontario introducing bill to ban sale of flavoured-tobacco to youth. The Toronto Star. 2013 Nov 13. http://www.thestar.com/news/queenspark/2013/11/13/ontario_bill_to_ban_sale_of_flavouredtobacco_to_youth.html.

